# Computer-Assisted Design Template Guided Percutaneous Radiofrequency Thermocoagulation through Foramen Rotundum for Treatment of Isolated V2 Trigeminal Neuralgia: A Retrospective Case-Control Study

**DOI:** 10.1155/2019/9784020

**Published:** 2019-03-03

**Authors:** Ran Wang, Ying Han, Lijuan Lu

**Affiliations:** Department of Pain Management, Nanjing Drum Tower Hospital The Affiliated Hospital of Nanjing University Medical School, Zhongshan Road 321, Nanjing, China

## Abstract

**Objective:**

Radiofrequency thermocoagulation (RFT) through the foramen rotundum has emerged as an alternative for treatment of isolated V2 trigeminal neuralgia. But puncture of the foramen rotundum is difficult and time-consuming. In current study, we introduced the application of a computer-assisted design (CAD) template to guide foramen rotundum cannulation. Meanwhile, we assessed its safety and efficacy in the treatment of isolated V2 trigeminal neuralgia.

**Methods:**

From November 2015 to August 2017, thirty-eight patients with isolated V2 trigeminal neuralgia were treated with computed tomography- (CT-) guided RFT through the foramen rotundum in our institution. All cases were reviewed, and patients were divided into the experimental group (*n*=17, puncture with a CAD template) and control group (*n*=21, free-hand puncture) according to the puncture method used. The puncture times, duration of puncture, and duration of operation were collected. The outcome of pain remission was evaluated utilizing the Barrow Neurological Institute's (BNI) pain score. Complications and recurrence of pain were also recorded. Data were compared between groups.

**Results:**

The rate of one-time successful puncture in the experimental group was obviously higher than that in the control group. Mean puncture times in the experimental group was fewer. Average duration of puncture and operation in the experimental group was also shorter than that in the control group. All patients experienced good pain remission (BNI Class I or II) postoperatively. At four follow-up points (7 days, 3 months, 6 months, and 12 months after operation), there was no significant difference in good pain relief rate between the two groups. Meanwhile, no significant difference was found in complications.

**Conclusions:**

CAD template is a safe and precise navigation instrument for RFT treatment of isolated V2 trigeminal neuralgia via the foramen rotundum. Therefore, this novel tool is worthy of clinical promotion.

## 1. Introduction

The idiopathic trigeminal neuralgia (ITN) is a common clinical condition characterized by recurrent, paroxysmal, and tortuous facial pain occurring in one or more divisions of the trigeminal nerve [1, 2]. As reported, prevalence ranges from 4 to 5 per 100,000, and ITN is one of the most excruciating pain syndromes that severely impairs the lives and work of patients [3, 4]. Drug therapy is the prior choice including carbamazepine or oxcarbazepine, but a multitude of patients cannot endure the side effects of drug tolerance and high-dose drugs [5, 6]. Quintessentially, these medically intractable individuals require invasive treatments including surgery, gamma knife radiosurgery, and interventional therapies [7–9]. Radiofrequency thermocoagulation (RFT) of the gasserian ganglion has long been applied in the clinical treatment of ITN. Cannulation through the foramen ovale (FO) is the most widely used approach [10, 11]. During this procedure, to avoid an inadvertent neurolytic block of the unaffected branches, sensory stimulation is always needed to locate the target neuron in the ganglion [12–14]. However, sometimes, the adjustment of stimulation in the ganglion was repeated many times which was a painful process. In order to solve this problem, selective V2 branch lesion through the foramen rotundum (FR) has been proposed [15]. This method demonstrated great advantages in terms of accurate ablation, but the cannulation of FR requires well-developed technology and familiarity of the anatomic structure.

Blind puncture could cause serious complications. Huang et al. [15] first introduced the technique of selective V2 branch ablation through the foramen rotundum. Their results confirmed the superiority of this technique in terms of safety and effectiveness compared with RFT through FO. Wan et al. [16] and Xue et al. [17] reported the application of RFT via the foramen rotundum for treatment of isolated V2 trigeminal neuralgia, respectively, under the guidance of CT and DSA. Their results verified the stable curative effect of this technique, but even under the guidance of imaging equipment, the process of puncture still requires multiple attempts before getting the external opening of FR. Repeated puncture increased the incidence of vascular injury and inadvertent puncture of maxillary sinus and infection. Multiple adjustments of the needle also increase the suffering of patients.

In recent years, the computer-assisted design (CAD) technique has been widely used for individualized guidance in the process of pedicle screw insertion [18, 19], dental implant [20], and maxillofacial surgery [21]. Based on these documents, we concluded that the CAD template could provide stable and accurate guidance for most puncture actions in various fields. However, rare research has applied this tool to the RFT procedure via the foramen rotundum. Here, we introduced the application of the CAD template to guide foramen rotundum cannulation for radiofrequency thermocoagulation. Thirty-eight patients with isolated V2 trigeminal neuralgia were reviewed to assess the safety and efficacy.

## 2. Materials and Methods

### 2.1. Patients

This study was approved by the review board of Nanjing Drum Tower Hospital (201810401). From November 2015 to August 2017, thirty-eight patients diagnosed with idiopathic V2 trigeminal neuralgia received RFT through the foramen rotundum in our institution. The diagnosis was based on the beta diagnostic criteria for ITN (International Classification of Headache Disorder, third edition) [1]. All the procedures were conducted by one operator (LJ Lu) in the CT room. All thirty-eight cases were reviewed and divided into the experiment group (*n*=17, puncture with a CAD template) and control group (*n*=21, free-hand puncture) according to the puncture method used in procedure. The demographic information of patients is depicted in Table 1.

### 2.2. Manufacture of CAD Template and Clinical Application

**Figure 1:**
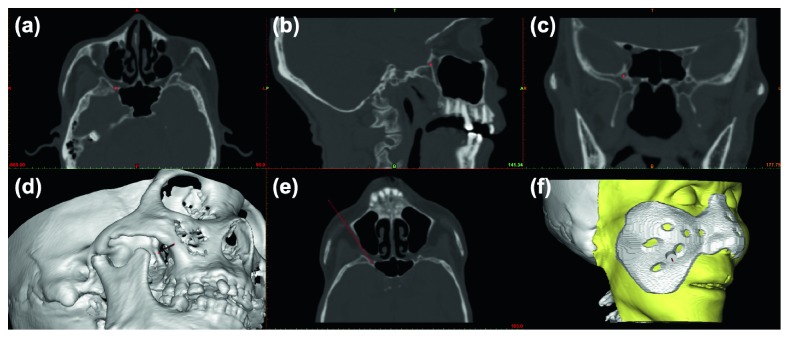
Progress of designing simulative trajectory and CAD template. We set the target at the external opening of the FR and confirm the right location (point) on the axial (a), sagittal (b), and coronal (c) views, respectively. A simulated puncture trajectory (cylinder) through the pterygopalatine fossa was then designed on the 3D image (d). The examination should be performed in the direction of the trajectory (e) with no bony structures, and paramount tissues obstruct the approach (line). After deciding the final trajectory, a template with a support bracket and a guide cannula was designed on computer software (f).

Manufacture of a CAD template requires slice CT (Brilliance TM CT, Phillip) scanning of the head (upper supraorbital arch to the chin, thickness as 1 mm). CT images (DICOM format) were imported into Mimics software (Materialize 17.0, Belgium), and three-dimensional images of the head were reconstructed. We set the target at the external opening of FR and simulate the puncture trajectory (in the process of the zygomatic, anterior to processus pterygoideus of the sphenoidalia, along the posterior lateral border of the maxillary sinus) through the pterygopalatine fossa [15]. The trajectory needed a slice of adjustment to avoid the bony structure or other crucial tissues before final confirmation. The intersection point of the approach and the skin was decided as the insertion point, and the distance between the target point and the insertion point was measured (Figure 1).

The template consists of two parts: a support bracket and a guide cannula. The support bracket was individually designed to fit patient's face perfectly to ensure stability. Meanwhile, when the patient's nose and zygomatic process were used as support structures, such a bracket should be large enough to cover these parts. The guide cannula was designed along a confirmed trajectory with an inner diameter slightly larger than the puncture needle. The personalized template of the designs was exported (in the format of STL) and imported into a three-dimensional printing (3D) printer (N2 type, Raise3D) for manufacture (Figure 1).

**Figure 2:**
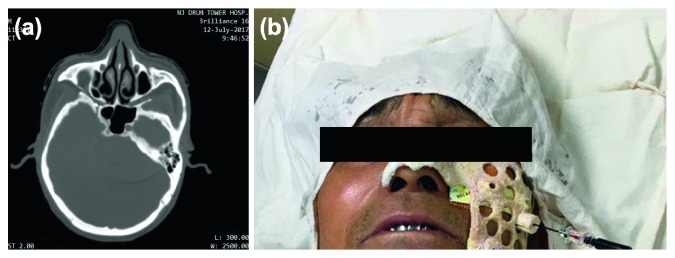
Clinical application of the template. After the process of skin preparation and draping, the template was placed on the patient's face stably and local anesthesia was given through guide cannula. Then, the needle was punctured to the measured depth through guide cannula. A CT scan was conducted to confirm the correct cannulation before the routine treatment process.

The CAD template was disinfected prior to clinical use. After the process of skin preparation and draping, the template was placed on the patient's face stably, and local anesthesia was given with 2% lidocaine via a guide cannula. Then, a 2 mm tip 22G radiofrequency needle was punctured to the measured depth through a guide cannula. A CT scan was conducted to confirm the correct cannulation before the routine treatment process (Figure 2).

In the control group, the cannulation of the foramen rotundum was conducted with the traditional method under the guidance of CT [16]. A lead panel was attached to the cheek of the symptomatic side as the marker for trajectory planning. After an oblique-coronal scanning, one specific CT image plane with the external opening of the FR and the FR canal was selected. We designed the puncture trajectory on this plane and measured the angle and depth of insertion. The entry point was marked on the patient's face. After the process of skin preparation and draping, local anesthesia was conducted with 2% lidocaine at the entry point. Then, a 2 mm tip 22G radiofrequency needle was punctured following the designed direction to the specific depth. A CT scan was conducted to confirm the correct cannulation. In both groups, routine RFT was performed after finishing cannulation and electrical stimulation test. The parameters of the RFT were set to 60°C for 120 s, 65°C for 120 s, and 70°C for 120 s.

### 2.3. Outcome Assessment

All medical history, surgery records, and follow-up records of these patients were obtained from the clinical information system. Images and the scanning time were collected from the picture archiving and communication system. The calculation includes duration of puncture (from the start of operation to the end of last scanning), duration of operation (from the start of skin preparation to the end of the RFT), and puncture times (equal to scanning times). We also assessed the therapy effectiveness using Barrow Neurological Institute's (BNI) score at each follow-up point (after operation, 7 days, 3 months, 6 months, and 12 months). Pain relief was classified as good (BNI Class I or II: no medication or no or only occasional pain) and poor (BNI Class III–V: drug need or drug failure, little pain, or severe pain) [22]. Complications like facial hematomas, numbness, leakage of cerebrospinal fluid, and muscle weakness were recorded.

### 2.4. Statistical Analysis

SPSS, version 23.0 (SPSS, Inc., Chicago, IL, USA) was used for the statistical analyses. Student's *t*-test was utilized to analyze differences in age, duration of disease, pain intensity, puncture times, and duration of puncture and operation. The chi-square test was utilized to analyze differences in gender, affected side, pain relief, and incidence of complications between groups. Statistically significant difference was identified by an exact *P* value < 0.05.

## 3. Results

In the experimental group, 15 of the 17 patients (88.24%) completed the cannulation of the foramen rotundum with one attempt. The remaining 2 cases (11.76%) took two tries to finish the puncture process. Only 4 patients in the control group underwent one-time success, and the average puncture times was 2.9 ± 1.1. The rate of one-time successful cannulation in the experiment was obviously higher than that in the control group (*P* < 0.05). Mean duration of puncture and operation in the experimental group was also shorter than that of the control group (puncture duration: 5.2 ± 0.9 mins vs 13.4 ± 5.7 mins, *P* < 0.05; operative duration: 14.4 ± 1.1 mins vs 24.1 ± 5.1 mins, *P* < 0.05) (Table 2).

All patients in both groups experienced good pain remission (BNI Class I or BNI Class II) postoperatively. The good pain relief rates of the experimental group at 7 days, 3 months, 6 months, and 12 months were 100%, 100%, 94.1%, and 88.2%. Two patients experienced recurrence of pain (BNI Class IV), respectively, five and ten months after operation. The rates of good pain relief at each follow-up point in the control group were 100%, 100%, 90.5%, and 85.7%, respectively. Three patients experienced pain recurrence (BNI Class IV), respectively at five, six, and nine months after operation. Five patients subsequently received repeated percutaneous RFT through the foramen rotundum and got satisfied postoperative pain relief (BNI Class I).

All patients experienced mild facial numbness in the second branch of the trigeminal nerve immediately after surgery, and this symptom gradually subsided within one to three months. There were 5 cases of facial hematomas in the control group and 1 case in the experimental group. Ice compress was used to attenuate this complication. There was no significant difference in facial hematomas incidence between the groups (5.9% vs 23.8%, *P*=0.196). No other serious complications were found.

## 4. Discussion

Percutaneous RFT of the gasserian ganglion through FO is traditionally used for treatment of medical intractable trigeminal neuralgia. Although this procedure has a high rate of successful pain relief (80%–98%) [15], the incidence of nonselective lesioning can reach as high as 16.7%–50% [12, 15, 23]. The inadvertent lesioning of the V1 or V3 branch may lead to numbness of the corresponding dermatome, motor weakness, absent corneal reflex, corneal keratitis, and even permanent vision loss. It is invariably arduous to get the target subbranches for the first attempt, especially when the affected branch is V2. Adjustment of the puncture direction is also scarcely feasible after the needle is inserted into the foramen ovale. As a result, withdrawal and reposition are required, which significantly increase the risk of dural puncture, infection, and intracranial bleeding. To solve this problem, percutaneous RFT through the foramen rotundum has recently been proposed as an alternative to the treatment of V2 trigeminal neuralgia [15–17]. The maxillary branch of the trigeminal nerve originates from the trigeminal ganglion and exits the skull through a foramen rotundum. The foramen rotundum is circular and relatively small in diameter (on average, 3.9 × 3.13 mm) [24]. After precise insertion into the external opening [15], the maxillary nerve can be easily accessed without further adjustment. At the same time, this method provides selective damage to the target branch and does not compromise the risk of V1 or V3 branching, demonstrating an edge over conventional therapies.

However, the opening of the FR is small, and its orientation is rarely parallel to the cannulation trajectory, making the puncture process more difficult than the cannulation of the foramen ovale [25]. Huang et al. [15] and Xue et al. [17], respectively, proposed the application of CT and 3D fluoroscope as a guidance in the procedure, but successful cannulation still needs several attempts, which increases the suffering of patients and risk of various complications. In our research, the average puncture times in the control group was 2.9 ± 1.1 (ranging from 1 to 4), which tally with the results reported in previous documents. As the trajectory passes through the pterygopalatine fossa, where the maxillary nerve is always accompanied by arteries, the probability of vascular injury and incident facial hematoma increases. Our results manifested that the incidence of facial hematoma in the control group was approximately 23.8%.

In recent years, with the development of CAD and rapid prototyping technique, an individualized template has been found applied in a variety of puncture procedures including denture implantation [26, 27], vertebral pedicle screw insertion [18, 19], active particles implantation [28, 29], and even joint replacement [30, 31]. Plenty of documents have confirmed the superiority of this novel navigation tool in enhancing the stability and accuracy. In our research, we applied this instrument to treat ITN via the foramen rotundum and found that the rate of one-time successful intubation in the experimental group was significantly higher than that in the control group. The average duration of puncture and operation in the experimental group was also shorter. Our outcomes demonstrated that the CAD template enjoys great advantage in improving the efficacy and accuracy of foramen rotundum cannulation.

In our study, all patients received satisfactory pain relief immediately after surgery. All patients experienced numbness in the second branch of the trigeminal nerve, and this symptom gradually subsided within one to three months. At the four follow-up points, there was no significant difference in good pain relief rates between the two groups. During the 12-month follow-up period, two patients in the experimental group and three in the control group experienced pain recurrence (BNI Class IV). Both patients achieved good pain remission after repeated RFT treatment through FR. Our results demonstrated that the efficiency of the RFT was stable as long as the needle tip was accurately placed at the external opening of the foramen rotundum. Wan et al. [16] found a recurrence rate of 10% during the mean follow-up period of 24.3 months. In the study of Xue et al. [17], the mean follow-up period was 14.74 ± 11.34 months, with an incidence of 36%. All these outcomes were similar to ours.

The preponderant complication is facial hematomas. The incidence of facial hematomas in the control group was higher than that in the experimental group. Even though the difference was not statistically significant, we believe that it could be increasingly obvious as the sample increases. In our research, we did not find other serious complications including intracranial bleeding, puncture injury of the V1 or V3 branch, and even cerebrospinal fluid leakage, as reported in previous research works [12, 15, 23, 32].

## 5. Conclusion

CAD template shows great potential in enhancing the safety and accuracy ablation of RFT for isolated V2 trigeminal neuralgia. This novel instrument deserves the attention of pain physicians, especially in resolving complex foramen rotundum cannulation. Meanwhile, prospective clinical studies are needed on a larger number of samples to further confirm our results.

## Figures and Tables

**Table 1:** Demographic information of patients.

Characteristic	Experimental group	Control group	*P*
Age (years)	67.7 ± 12.9	65.8 ± 13.4	0.661
Gender		0.796	
Female	9	9	—
Male	8	12	—
Course (years)	3 ± 2.9	2.8 ± 2.7	0.827
Side			0.973
Left	8	10	—
Right	9	11	—
VAS before operation	8.1 ± 1.1	8.1 ± 1.0	>0.999

**Table 2:** Comparison of data on the efficacy of two sets of intubation.

	Experimental group (*n*=17)	Control group (*n*=21)	*P*
Rate of one-time successful cannulation	88.24% (15/17)	19.05% (4/21)	<0.01
Puncture times	1.1 ± 0.3	2.9 ± 1.1	<0.01
Puncture duration	5.2 ± 0.9	13.4 ± 5.7	<0.01
Operation duration	14.4 ± 1.1	24.1 ± 5.1	<0.01

## Data Availability

The data used to support the findings of this study are available from the corresponding author upon request.
